# The politics and practice of initiating a public health postgraduate programme in three universities in sub-Saharan Africa: the challenges of alignment and coherence

**DOI:** 10.1186/s12939-020-01163-x

**Published:** 2020-04-09

**Authors:** Woldekidan Kifle Amde, David Sanders, Mohsin Sidat, Manasse Nzayirambaho, Damen Haile-Mariam, Uta Lehmann

**Affiliations:** 1 School of Public Health, University of Western Cape, Cape Town, South Africa; 2 Department of Community Health, Eduardo Mondlane University, Maputo, Mozambique; 3 School of Public Health, University of Rwanda, Kigali, Rwanda; 4 School of Public Health, Addis Ababa University, Addis Ababa, Ethiopia

**Keywords:** Capacity development, Complexity, Internationalization, Programme champion, Public health training, South-south cooperation

## Abstract

**Background:**

In-country postgraduate training programme in low and middle income countries are widely considered to strengthen institutional and national capacity. There exists dearth of research about how new training initiatives in public health training institutions come about. This paper examines a south-south collaborative initiative wherein three universities based in Ethiopia, Rwanda and Mozambique set out to develop a local based postgraduate programme on health workforce development/management through partnership with a university in South Africa.

**Methods:**

We used a qualitative case study design. We conducted semi-structured interviews with 36 key informants, who were purposively recruited based on their association or proximity to the programme, and their involvement in the development, review, approval and implementation of the programme. We gathered supplementary data through document reviews and observation. Thematic analysis was used and themes were generated inductively from the data and deductively from literature on capacity development.

**Results:**

University A successfully initiated a postgraduate training programme in health workforce development/management. University B and C faced multiple challenges to embed the programme. It was evident that multiple actors underpin programme introduction across institutions, characterized by contestations over issues of programme feasibility, relevance, or need. A daunting challenge in this regard is establishing coherence between health ministries’ expectation to roll out training programmes that meet national health priorities and ensure sustainability, and universities and academics’ expectations for investment or financial incentive. Programme champions, located in the universities, can be key actors in building such coherence, if they are committed and received sustained support. The south-south initiative also suffers from lack of long term and adequate support.

**Conclusions:**

Against the background of very limited human capacity and competition for this capacity, initiating the postgraduate programme on health workforce development/management proved to be a political as much as a technical undertaking influenced by multiple actors vying for recognition or benefits, and influence over issues of programme feasibility, relevance or need. Critical in the success of the initiative was alignment and coherence among actors, health ministries and universities in particular, and how well programme champions are able to garner support for and ownership of programme locally. The paper argues that coherence and alignment are crucial to embed programmes, yet hard to achieve when capacity and resources are limited and contested.

## Background

This paper is part of broader study that explores a multi-faceted south-south collaboration among four academic institutions in sub-Saharan Africa, to strengthen national capacity towards generating much-needed leaders to spearhead workforce development/management [1–3]. The paper examines one component of the collaboration, the initiative to introduce and embed a postgraduate level training programme focusing on health workforce development/management in three universities in sub-Saharan Africa (Ethiopia, Rwanda, and Mozambique) by drawing from the experience and expertise in the field from another university in South Africa. This paper seeks to generate insight into the complexity of this process, which navigated contestation of priorities and alignment among different stakeholders.

Shortages of human resources for health (HRH) are particularly pronounced in sub-Saharan Africa. Weaknesses in planning for HRH needs contribute to this situation. The lack of leadership capacity for HRH and the absence of local leadership development programmes in the region partly underlie this crisis [4–7]. Literature depicts the importance of having in-country postgraduate training programmes in Low and Middle Income Countries (LMICs) as a critical intervention to strengthen institutional and national capacity to address health system challenges. Such initiatives are credited to reduce cost of training, improve access, enhance curriculum relevance, curb brain drain, and promote sustainability of programmes [6, 8, 9].

Neufeld and Johnson (2004) in their review of supply side studies noted the lack of leadership development training programmes in the Global South, and the dominance of institutions in the Global North in the few leadership development programmes in/for LMICs. The authors further highlighted the disproportionate emphasis given to developing individual capacity as opposed to institutional capacity such as infrastructure, curriculum and teaching capacity development, and incentives for staff retention and motivation [4]. In line with this, health training institutions in the Global South are fraught with challenges related to shortages of funding, academic and support staff, teaching space, and capacity to develop training materials and curriculum [10–13].

Operating under such circumstances, training institutions have become contested spaces in the globalization (and marketization) of health professions education since programme development in the South becomes commercially interesting for higher education institutions in the North, with large sums of money available. Such contestation around programme priorities, and the competition for financial and very scarce human resources plays out in different ways, and in complex relations between northern and southern academic institutions in this regard [14].

A growing body of literature on capacity development emphasizes its complexity, as it often involves ill-defined non-linear processes that bring into interaction multiple actors with diverse interests and priorities [15–22]. It is now well established in the literature that sustainable capacity development requires close attention to these complexities in planning, implementation and research, and exploring the factors that nurture or undermine capacity development within and across the different and interacting levels [15, 17, 23–30]. In other words, it is imperative to pay heed not only to the technical aspects, but also the politics of capacity development and its institutional sustainability [20].

### Emergence of the south-south collaborative initiative

Partnership, through its multiple variants, is recognised as one of the mechanisms to bring about development. South-south cooperation is one form of development partnership among a wide range of actors located in LMICs [31, 32].

In year 2009 three of the universities (from Ethiopia, Rwanda and Mozambique) set out to develop a postgraduate programme focusing on health workforce development/management with technical support from the South African university. The support included providing educators from these universities a masters level training in health workforce development/management, development and adaptation of teaching resources, and sharing experience through workshops on delivery of open and distance teaching modalities. Funded by the World Health Organization (WHO) the initiative sought to respond to the twin challenges facing public health training universities of ‘building human resources capacity in ministries and health services while alleviating and improving their own capacity constraints’ [2]. The financial support from WHO sought to enable the implementation of the aforementioned activities led by local programme champions who oversee the development and integration of programme in the three universities [2]. This meant ‘fitting’ the programme into the landscape of programme offerings in each of the universities. The programme had support from the health ministry in each country (Rwanda, Mozambique and Ethiopia) and leadership of the implementing universities. The collaborative project ran from 2009 to 2015.

Overall, the initiative has distinct features of south-south cooperation, which is considered a viable mechanism to facilitate capacity development in developing countries [33, 34] by enabling exchange of knowledge, experience, and resources among Southern partners [35, 36]. This initiative presented an opportunity to explore factors that influence process and outcome of a capacity development initiative across multiple institutional and national contexts, and to explore how contextual or relational factors assisted or undermined coherence and alignment.

## Methods

We used a qualitative case study design [37–40]. This research design suits the complex nature of the phenomenon under investigation and helps explore the context and the discrepancy between what was envisaged and what materialized [38, 39]. Purposive sampling was used to recruit study participants (*n* = 36) including 17 academics located at the three public health training universities; 13 staff of health ministries in Mozambique, Rwanda and Ethiopia; and six representatives of external development or training partner institutions. The selected participants were directly or indirectly involved in the design or implementation of the collaborative partnership to introduce postgraduate programme in health workforce development in the three universities. Programme champions, who were academics located in universities were tasked with the responsibility of championing programme. The rest of the participants were directly or indirectly involved in the design or implementation of the collaborative partnership to introduce postgraduate programme in health workforce development in the three universities. Their participation includes taking part in collaborative curriculum development workshops and exchange of experience, participating in the periodic general partnership meetings, or be part of a university or health ministry structure that developed, reviewed, approved, or implemented the programme. Table 1 presents distribution of participants by institutional location and gender.

**Table 1:** Characteristics of participants

Institutional affiliation	
University A	4
University B	6
University C	7
External development/training partners	6
Health ministries	13
Gender	
Men	28
Women	8

Semi-structured interviews were held with the participants between June 2014 and March 2015 in their respective contexts. The first author held 34 interviews in person, and two interviews telephonically. The interviews primarily explored perceptions and experiences of actors over a range of contextual and relational factors that mediate the process and outcome of the partnership to initiate a public health postgraduate programme in health workforce development. The semi-structured interviews explored the following broad issues:

- State of capacity for health workforce development at individual or institutional level;
- Internal conditions including programme implementers and targets, institutional context and processes;
- External conditions or factors in the broader context that have bearing on the process and outcome of the intervention;
- Stakeholders, partnership, programmes and resources related to the intervention;
- Process of implementation of various components of the partnership to develop capacity in health workforce development; and
- Mechanisms that enable or constrain the intervention.

The interviews were mostly conducted in English with participants from Rwanda and Mozambique, who completed tertiary education and had good command of English. The interviews with participants from Ethiopia was done using Amharic, the country’s official language. All interviews were audio recorded, with the permission of participants, which were then transcribed verbatim. The first author transcribed all the English interviews and translated the Amharic interviews.

We gathered supplementary data through review of project documents including proposals, agreements, reports and email correspondence. Unstructured observation was another source of data. First author took part in the implementation process, attended meetings and workshops, and carried out field visits in the collaborating universities across the three countries. Hence, observational information, own reflections and analytical memoranda regarding activities, processes, and interactions were very integral to the analysis process.

**Fig. 1:**
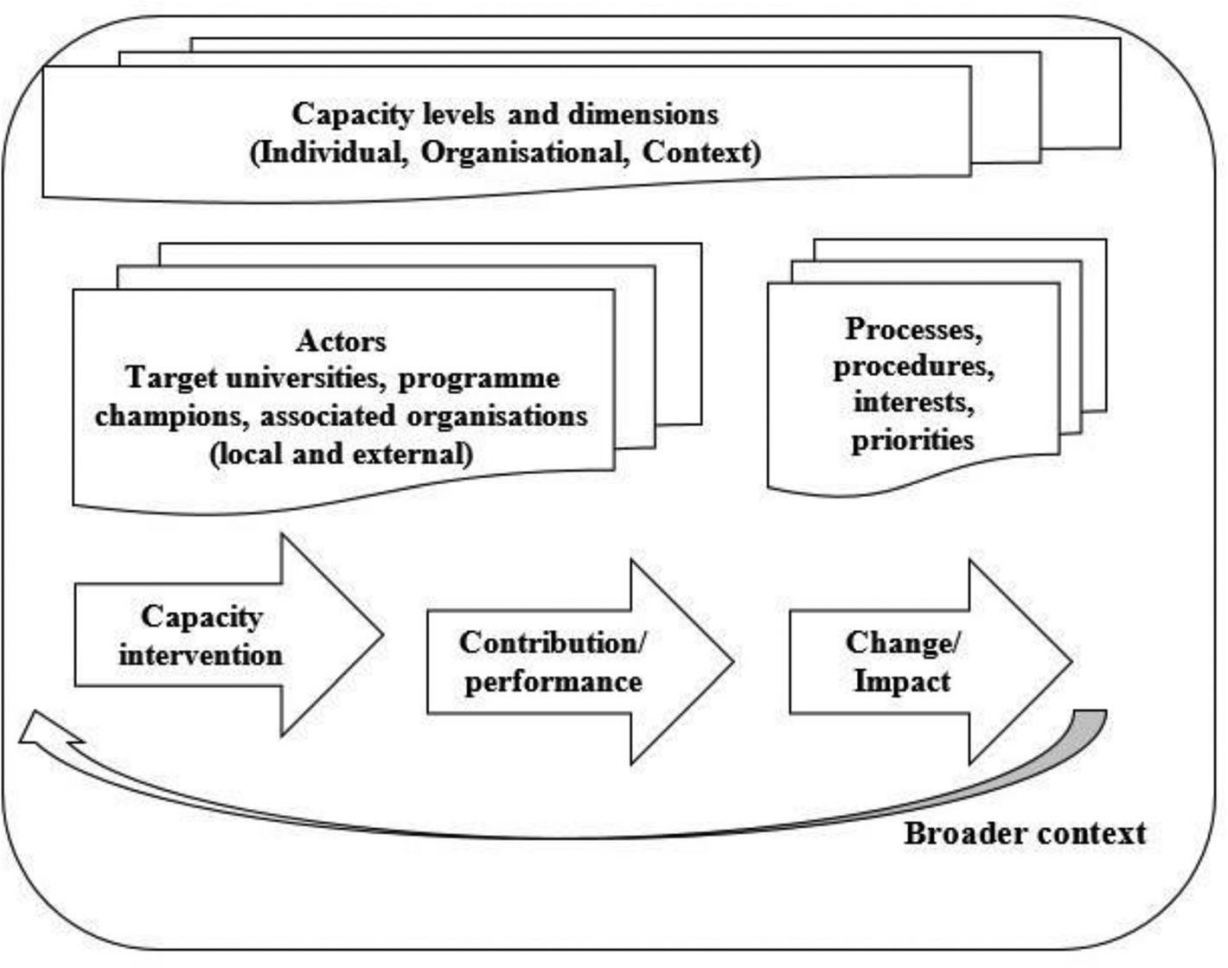
Conceptual framework for analysing capacity development interventions

Triangulation and reflexivity were applied to ensure rigor and trustworthiness of the research findings [40–42]. We analyzed the data thematically. Through an iterative process, the researcher open coded the transcripts manually with a focus on describing the different data segments. The induced codes were then grouped into more analytical categories/themes (programme introduction modalities- regular and special, various roles and characteristics of actors in programme introduction) that spoke to the conceptual framework drawn from the literature that presents capacity development as a complex systems phenomenon embedded in interactions across multiple domains – individual, institutional, and context [15, 26, 29, 30] (Fig. 1).

The framework seeks to map the change process inherent in capacity development such as this and the multiple mediating processes and actors. It identifies three iterative phases of the changes process: capacity intervention (collaborative partnership and associated resources and expertise), contribution/performance of capacity (curriculum integration and training roll out), and effecting change/impact (development of critical mass, and improved national leadership and training capacity in the field). This transition is not guaranteed as capacity generated may remain untapped and may not lead to improved performance or change [43].

According to the framework, the process and outcome of the collaborative intervention to integrate curriculum and roll out training should be understood by foregrounding this dynamic process in the web of capacity levels and dimensions encompassing actors and processes operating at multiple levels (individual, organisational, and environmental), and surfacing the underlying mediating contextual and relational factors.

Permission to conduct the research was obtained from the Senate Research Committee of the University of the Western Cape, South Africa, which is the IRB/ethics committee responsible for development and monitoring of all university ethics policies and procedures. Confidentiality and anonymity of participants were ensured by removing any identifying information, and using systematic codes to refer to institutions and respondents [44]. The three implementing institutions are referred from hereon as University A, University B, and University C.

## Results

This section presents findings of the research under two broad categories that focus on actors and processes. Emerging patterns and themes with respect to initiating a postgraduate public health programme in the three universities are presented as they relate to the prominent actors (roles and characteristics) and pertinent processes of programme introduction (regular and special programmes, and process of curriculum approval).

### Mapping practices and processes of programme introduction

The three universities have a wide variety of programmes, with distinct organizational processes regarding how such programmes are initiated, implemented, and sustained. Two broad models of programme introduction prevail in these public health training institutions, i.e. regular and special (See Table 2 below).

**Table 2:** Modalities of postgraduate programme initiation

Features	Special	Regular
Admission criteria	Accommodative/flexible	Strict/stringent
Selection	Done by training institution with health ministry	Selection done by University, as per rules of education ministry
Incentives to staff	Additional payment	No additional incentive
Period	Weekends, after hours/evening	Day, regular
Staff	Existing or guest staff	Existing or additional staff needed
Sustainability	Not guaranteed, some evolve to regular with additional staff	Sustainable

Both routes of programme introduction require collaboration with multiple actors within and outside the training institution to fulfil requirements related to feasibility, relevance and quality of curriculum. Regular programmes are fully embedded in the institutional structures and enjoy health ministry’s approval and support, and have a longer life span. Special programmes are introduced through temporary technical and financial arrangements (with respect to funding, level of entry and selection of trainees), with the expectation of gradually transitioning into regular programmes and thus by definition have a temporary life span. Table 3 below summarizes the varying practices in the universities with respect to introducing and sustaining postgraduate programmes.

**Table 3:** Modalities of postgraduate programme introduction across universities

Institutions	Special programme	Regular programme
University A	All postgraduate programmes	
	Generates additional incentive for staff	
	Initiated by training institution with/without support from MOH or external development or training partners	
University B		All postgraduate programmes
		Initiated by MOH or training institution with/without support from external development or training partners
		May require hiring additional staff, if institution operating at capacity, but no additional incentive for staff
University C	Some Postgraduate programmes	Most postgraduate programmes
	Initiated by MOH with external development or training partners	Initiated by MOH, training institution
	Involves incentives for staffs	May require hiring additional staff, if institution operating at capacity, but no additional incentive for staff

In the case of University C both regular and special models are common ways of introducing postgraduate programmes. In University B the regular model is the most common, and the special arrangement happens often for certificate or short courses. In University A all postgraduate programmes have a special status.

Ministry of Health is insisting that we start [a new programme] right away. But we responded that we wouldn’t start in a rush, before clarifying how it is going to be operationalized. Is it a regular programme, or what? If it is regular, the registration is done through the university. If it is to be done as special… then you organize special classes, Saturdays, Sundays, or evening. If it is going to be special, then we need to make provision for staff, you can’t just ask them to teach. Time is precious.

[P17, University C]

Prior to introducing a programme, training institutions have to fulfil curriculum requirements, which vary across institutions, with some more protracted than others. With the growing number of programmes and in the context of shortage of capacity in the institutions, stringent curriculum approval processes are put in place to ensure introduction of only priority programmes, e.g. Generic MPH and Field Epidemiology.

With respect to the new training programme on health workforce development/management, none of the three universities had such a programme, which is also distinct from the generic MPH running in all the universities at the time (See Table 4 - Inventory of programmes being offered in the universities at the time of the intervention).

**Table 4:** Inventory of programmes running at the institutions at the time of the intervention

University A		University B		University C	
Postgraduate Programmes	Mode of delivery	Postgraduate Programmes	Mode of delivery	Postgraduate Programmes	Mode of delivery
Health systems	Speciality track, special programme	Public Health	Face to face, regular, evening	Public Health	Face to face, regular Evening, Special programme
Human Resource development	Speciality track, special programme	Epidemiology	Face to face, regular, evening	Reproductive Health track	Speciality tracks, regular
Disease prevention	Speciality track, special programme	Field Epidemiology and Laboratory Management	Blended, regular	Health Service Management track	Speciality tracks, regular
Public Health and Bioscience	Special programme	International Health Management	Special	Epidemiology	Speciality tracks, regular
		Health and Hospital Management	Blended, Regular	Environmental Health	Speciality tracks, regular
			Regular	Field Epidemiology	Special programme
				Public Health Nutrition	Regular programme
				Hospital Administration	Special programme
				Health Informatics	Special programme
				Hospital and Health Care Administration	Special programme
				Health Economics	Special programme

University A effectively integrated the new curriculum in 2014, translating curriculum and teaching resources, and mobilizing resources from external partners to this end. The institution publicized the programme among staff and leadership of the health ministry, which sponsors trainees. At the time of data collection, over 70 trainees of multiple cohorts have been enrolled in the programme.

In University B and C, the envisaged programme did not materialize. The institutions needed an explicit expression of interest, memorandum of understanding, to support integration of programme from the health ministry either through providing funding for the special programme, or making necessary investment on personnel and infrastructure for it to become a regular programme. The south-south partnership did not have funds for these required investments. Trained staff in the short term used the teaching materials designed for the programme to strengthen existing programmes.

In the sections that follow, we unpack these varying developments across universities and explain the contrasting success in implementation of the programme between University A, and the two other universities (B and C).

### Mapping actors, agendas and interactions

The three public health training universities interact and collaborate with a range of external development and training partners, as well as local actors who influence the nature, scope, and success of their own engagement in the countries and beyond, all in the course of implementing their mandate, which encompasses teaching and learning, research, and extension/community outreach service. Table 5 illustrates the various prominent stakeholders in the process of introducing training programme in academic institutions.

**Table 5:** Stakeholders and roles in the process of introducing postgraduate programmes

Stakeholders	Characteristics/role in programmes
Development partners (e.g. USAID, CDC, UNFPA, WHO)	Primary donors
External training partners (e.g. JEPIEGO, TULANE, MSH, YALE, RENNES)	Technical cooperation/ Implementers
Public Health training institutions	Implementers
Ministry of Health	Current/future employer of trainees
Ministry of Education	Regulator/Owner of training programmes
Staff/faculty at local training institution	Implementers
University/college/faculty	Parent institution, regulator
Programme champions	Boundary spanners/gate keepers/Change agents

These actors that exert distinct influence to enable or constrain the process (either through support, opposition, or inaction) include academics who assume the role of championing the programme, the training institution, relevant government institutions namely health ministry, and external development or training partners.

Any of the aforementioned actors can initiate programme introduction, but the success of the initiative and its sustainability require support from all or most of these actors, which depends on alignment of their respective agendas. The following part of this section describes these actors and their roles and relationships in the context of introducing postgraduate programmes.

### Health ministry

The health ministry is a custodian of the health sector and prescribes strategic direction and programmes to public health training universities. The health ministry defines which training programmes are relevant to address national health priority, and outlines core competencies. Even though universities are under the governance of the education ministry, the health ministry assumes the above roles due to its superior expertise of the needs and resources in the sector.

The health ministry often leads the role of initiating new public health postgraduate programmes in the institutions, by working with/through the three institutions, academics, or external partners. The ministry also initiates programmes particularly when those programmes are considered basic in the context of national health priorities such as Masters in Public Health, or Masters in Field Epidemiology.

While in theory academics, training institution, or external partners could initiate a training programme in the three universities, key informants made it clear that successful introduction or sustainability relies on the buy-in and ownership from the health ministry. One senior academic staff explained,

… Our [University B’s] training should contribute to resolve a given problem in [the country] or in the region…. We are free when we identify the need, we can also suggest the introduction of a given programme. But … we are [not] able to start a programme without the approval of the Ministry [of Health].

[P52, University B]

The role of the health ministry in initiating the curriculum varied across the countries, with the ministry playing a more active role in University B and University C than in University A. In the case of University B and University C, it was reported that the ministry is the main stakeholder in training needs assessment and providing a required list of competencies.

In the course of introducing the training programme on health workforce development/management, health ministries in each country endorsed the proposed programme as relevant and expressed support in the early stage of the collaboration in 2009. However, none of the universities received any tangible support from the ministries during the course of the collaboration (2009–2015), which undermined programme introduction particularly in the University B and University C. University A had somewhat better leverage of introducing programme, possibly due to the relative autonomy the university has in its relation to the health ministry.

The lack of support from ministry of health in University B and University C can be attributed to multiple factors. One of the factors is turnover of programme champions based in the universities or turnover of leadership at ministry of health, which resulted in the loss of implementation momentum or loss in institutional memory.

… It [health ministry] did have enough information [about programme]. … We [the local project team] have tried to communicate this with the ministry of health. But since the first communication …there has been a turnover of three ministers.

[P50, University B]

This was further complicated by the advent of parallel processes in the two universities to introduce similar programmes by the health ministries with the backing of other external partners from the North.

### Public health training universities

Like other academic institutions, the three universities have the mandate to train the next generation of public health professionals to meet national health priorities, but rely on ministries of health for strategic guidance and support when it comes to the programmes they offer.

While all the three universities had complex relationship with the health ministry, these complexities presented differently and impacted on project outcomes in different ways. University A has a relatively high level of autonomy from ministry of health when it comes to introduction of new postgraduate programmes. The programmes are open to private applicants and students working in non-government organizations while staff of the health ministry remain the main clients.

[The postgraduate programmes] emerged in a decentralized [fashion]. Faculties came up with the proposal, waited for approval and started the programme. Neither administrative nor academic management [of the programme] is centralized.

[P31, University A]

In University B the health ministry exerts a great deal of influence when it comes to programme introduction, and sends most of the trainees.

When we started this school [in 2001], it was with the objective … to produce the health professionals for Ministry of Health [which] pays their tuition fees. … [Currently] Most of our students are from Ministry of Health.

[P52, University B]

University C has very close relation with MOH. Students mainly comprise ministry staff and academics from other universities. Lack of alignment in the interests of the health ministry on one hand, and the university and academics on the other hand (over issues of funding or selection of trainees) put a strain on their relationship. This was evident with two prominent but parallel processes of programme introduction prevailing in the university, regular and special. Key informants in University C reported that lately MOH is pushing for regular programmes, as it considers special programmes as unsustainable, as special programmes require additional financial incentives, on top of salaries, for those academics involved in teaching or coordinating the programme. The opportunity cost for academics who participate in new programmes without additional remuneration, as described in a related publication, is time away from engagement in external multiple job holding practice, which offers financial and professional benefits [1]. The training institution on its part claim to operate at full capacity and resist hosting new programmes without the necessary investment in personnel and infrastructure. A senior academic drew attention to the mismatch between MOH expectations and investment towards building capacity of training institutions,

[I] can't really say the support from MOH to university is high or strategic. Because it changes when there is turnover. It also gives you the programme, and does not give you anything [else]. Except for [giving us the] go ahead. No financing.

[P20, University C]

Conversely, MOH representatives contend that the training institution is performing below capacity and should accommodate new programmes that would meet the health workforce need of the sector. One government official described the practice of running special programmes or providing incentives to staff associated with the programme as perverse:

… If it [training programme] is project based [special], … it turns teachers into rent seekers [and] … it won’t have sustainability. It should be part of the [regular] system and integrated, and necessary capacity building, equipment, books should be fulfilled like offering training to teachers. That is when capacity building becomes sustainable.

[P5, University C]

### Programme champions

The initiative to introduce the new programme on health workforce development/management had designated programme champions, who were senior academics in the training institutions. In University A the programme champion, a senior academic of the training institution, engaged with MOH about the programme, and was committed to spearhead the implementation of the programme in the university. The university initiated the health workforce development/management programme, with the programme champion ensuring that curriculum approval within and outside the institution was accomplished. Curriculum approval went through various processes at multiple levels (see Table 6 – Process of curriculum approval).

**Table 6:** Curriculum approval process across universities

Process of curriculum approval		
University A	University B	University C
Department	Department	Department
Postgraduate Council, Faculty	School council	School
Scientific Council, University	Academic senate	College
Postgraduate committee, Scientific Directorate, university	Board of directors of university	External reviewers
Academic Council, university	Ministry of Education	Graduate Councils/Senate
University council	Ministerial cabinet	
Ministry of Education		

In University C the programme champion, a senior academic staff, faced challenges to advance the programme integration. The implementation process stalled for long periods in the absence of explicit expression of interest to support the programme from the MOH [either provide funding if special programme, or make necessary investment on personnel and infrastructure if regular], and the lack of adequate funding in the existing partnership with external partners to support investment in infrastructure or teaching personnel. This also coincides with the turnover of the programme champion and initiation of a parallel process to introduce a similar programme in the institution with support from the health ministry and external partners. Despite being able to initiate the curriculum approval process, the programme still needed to get approval from the college, external reviewers, and graduate council/senate.

In University B, two senior academic staffs were tasked to champion the introduction of the programme in the institution. They initiated the curriculum development process, but they stopped short of taking the curriculum through all the required levels of approval. This coincided with a change in leadership at MOH, lack of explicit interest and support from MOH, turnover of one of the programme champions, and end of project funding.

A notable difference with respect to programme champions across the three universities is that the designated programme champions in University B and University C vacated their senior leadership positions in the university during the course of the intervention. With the departure of the senior programme champions, institutional memory about the programme, and the momentum and potentiality of securing MOH buy-in were undermined. The programme champion in University A was actively engaged throughout the course of the intervention.

### External development and training partners

External partners work through or with local actors namely academics, training institutions, or government institutions to initiate programmes to address institutional and national capacity needs in certain areas, by offering financial or technical support. Some of the external development partners (e.g. United States Agency for International Development, Centers for Disease Control, United Nations Population Fund, WHO) and external training partners (e.g. Johns Hopkins Programme for International Education in Gynecology and Obstetrics, Tulane University, Management Sciences for Health, Yale University, University of Rennes) were influential in one or more of the universities.

Engagement of the three universities with external partners can generally be characterized as fragmented. The lack of coordination has led to competition, duplication, and loss of capacity gains, whereby academic institutions or the health ministry choose one initiative over another, or allow both to co-exist, instead of seeking synergy or harnessing partnerships and support around similar initiatives.

Northern training partners dominate the partnership space in the three universities and with MOH (in terms of resource or influence) compared to Southern training partners. In the case of University B and University C, there were reports of parallel processes to introduce a similar programme in human resources management in the institutions in collaboration with Northern external collaborates and led by different academic programme champions within the universities. In the two countries, there were inclinations towards collaborating with Northern external training partners, which are well resourced.

A key informant expressed exasperation about proliferation of similar initiatives often led by Northern external training partners as a case of, “…. the funder tail wagging the capacity development dog.” [P22] Recounting the ambivalence surrounding the scenario in University C in relation to decision about the choice of partners or programmes, a key informant stated,

… I can see that [this south-south] programme is the first [to be initiated]. … How do we go about it? Do we merge [it with a similar programme initiated with northern partners]? … were we supposed to accept [just this programme] … or did we do the right thing [to choose the programme supported by northern partners]?

[P17, University C]

**Fig. 2:**
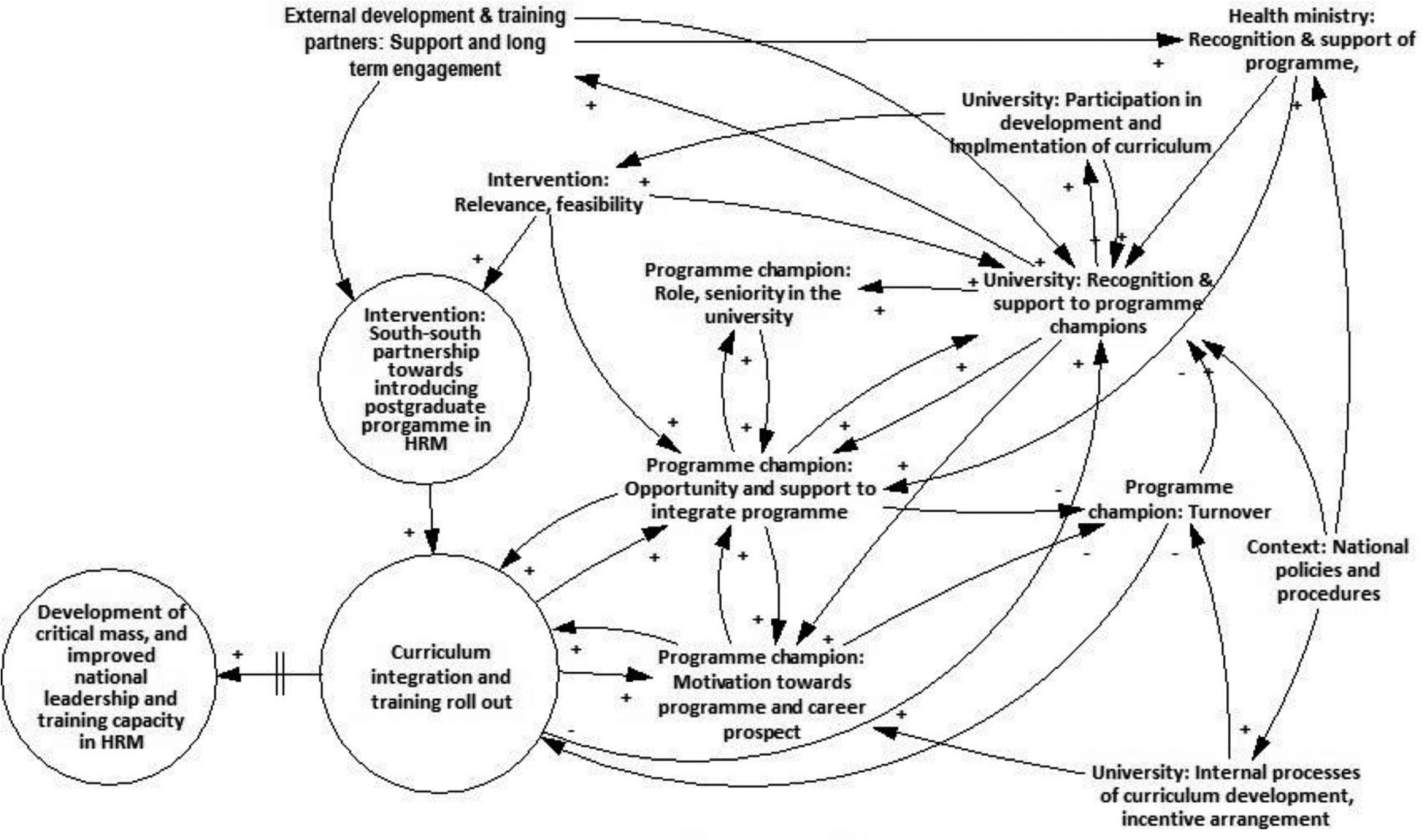
Multilevel factors mediating curriculum integration and training roll out

The figure below summarizes the aforementioned analysis, and highlights the prominent actors, processes and mechanisms that influenced the introduction of the postgraduate programme in public health in the three universities (Fig. 2).

The above figure shows the link between intervention, and associated resources and expertise, towards introduction of a postgraduate programme, and state of curriculum integration and training rollout in the target universities, and future changes in terms of development of critical mass of leaders, and improved national leadership and training capacity. It is evident in the above analysis that this transition is mediated by dynamic set of factors associated with multiple actors (external development and training partners, universities, programme champions, health ministries) and contextual factors within the university and beyond. Hence, curriculum integration or training roll out is contingent on seniority, role and motivation of programme champions, and opportunities and support at their disposal. Factors associated with the target universities and health ministries including recognition and support of programme or its champions, and incentive arrangements impact on motivation and opportunities of programme champions to spearhead integration of programme. Contextual factors such as national policies and procedures have a bearing on successful implementation of intervention. Interaction across these individual, institutional and context level factors further determine retention of programme champions in the target universities. Overall, the degree of alignment across these multiple actors and processes enable or constrain the success of the capacity development intervention resulting in the short-term outcomes of partial or non- integration in two universities (B and C), and full integration in University A.

## Discussion

Baser and Morgan (2008) in their report entitled “Capacity, Change and Performance” emphasized the need for coherence in the context of complex capacity development initiatives that are characterized by multiple interrelated causes and potentiality of unintended outcomes [15]. The authors further commented, “Capacity development was not just a technical exercise in achieving better development performance. It was, in practice, a process that allocated authority, opportunity, resources and security to some and not others” [15].

Evident in our analysis and discussed further in the sections below is the differential and dynamic experience of implementing the initiative across contexts owing much to the unpredictable interaction among multiple actors that is embedded in contestations over issues of programme relevance, feasibility, and actors’ roles.

### Confluence of power relations, and need for coherence

When it comes to programme introduction, the study established that success of initiatives depends on the degree of alignment achieved among the agendas and expectations of the various actors within and outside the institutions, who have varying influence on availability of resources for programmes, their legitimacy, implementation, and sustainability. The process is embedded in contestations over issues of programme feasibility, relevance, and need. The financial feasibility of programmes is contested between institutions and MOHs, institution and external partners, or institution and academics. The issues include academics’ workload and financial incentives, the training institution’s capacity to rollout the programme, or resources at the MOH’s disposal to support the initiative in the institutions.

With respect to programme relevance and need, in all the three countries MOH has the discretion in deciding whether a programme meets a national priority, which is also influenced by the national and international priorities and interests, through the roles played by external partners. Future research needs to examine more closely the perception about global or local orientation of existing programmes, and practice of priority setting within the health ministries with respect to core competencies or training programmes.

Cancedda et al. (2015), based on assessment of training initiatives originating through North-South collaboration in sub-Saharan Africa, recognized the confluence of factors influencing such initiatives and underlined the importance of, among others, adaptability, local ownership and funding, coherence between training and country health priorities, long term engagement, and integration and continuity of programmes [10]. Our analysis of the experience of the three universities resonates with these lessons, even though in the very different context of a south-south collaboration. The successful integration of programme in University A is down to convergence of the aforementioned issues, while University B and University C fell short of embedding programme due to lack of the crucial elements namely, inadequate ownership or funding, and short-lived engagement of key actors like programme champions and MOH.

### Duplication of efforts, need for harmonization

One of the challenges observed, in the case of University B and University C, which undermine programme introduction or sustainability was the lack of coordination of efforts, and the resulting presence of parallel initiatives. There were reported instances of local and external partners of public health training institutions working in silos in their respective collaborations with the in the case of University B and C, which pose challenges of competition, duplication, and resource wastage. On the same note, a study drawing from four major initiatives in LMICs, highlighted the barriers in on-going practices of training initiatives for health professionals. In the face of poor coordination and communication about these training initiatives among stakeholders, including MOH and regulatory bodies, ‘low-income countries have been on the receiving end of a disorderly patchwork of small-scale, insufficient quality, short-term, and unsustainable health professional training initiatives … created unnecessary gaps or overlaps in resources, and failed to help meet long-term national health workforce needs’ [10]. The authors argue for a more prominent role by health ministries to coordinate these efforts [10]. However, a concern with such an arrangement, which represents the position of health ministries, is the widely prevalent practice of choosing and supporting Northern partnership over Southern ones, and crowding out of some programmes that are not considered high priority by MOH.

The exclusive focus of universities on so called basic programmes, a common pattern in MOH’s programme selection, can be detrimental to the needed mix in the range of public health professionals, in the context wherein only a few public health programmes and training institutions exist in the countries. This can also undermine internationalization of academic institutions with respect to the diversity of global programmes in public health. What is evident in this study is that universities or academics lack autonomy in this regard.

Contestation between health ministries and universities over issues of quality, relevance or alignment of training programmes with national priorities can be located in the debate about internationalization of higher education in Africa, wherein institutions engage in exchange of global knowledge and know-how towards executing their academic and research mandates [14, 45]. Academic institutions partnership with external partners can be characterized as having an internationalization agenda, whether it is training of academics abroad or adapting a new programme. Internationalization, which has an outward approach and emphasizes quality and standard, may not be aligned well with an inward focus of meeting local priorities. Jowi (2009) noted that the internationalization of higher education in Africa is motivated mainly by the need to revamp the academic and management capacity of the institutions, in the face of inadequate support from government. This explains predominance of well-resourced northern partners in internationalization initiatives in Africa compared to partners from south or the African region in particular despite a growing interest in the later types of cooperation [14]. Finding coherence in the internationalization agendas is thus a daunting challenge that higher education institutions and governments in Africa grapple with in the context of lack of clarity regarding roles that the various actors play in the implementation of internationalization [46].

### Need for strategic investment to support south-south cooperation of universities

Despite the increasing demands put on universities to meet national health workforce needs, it was apparent in this research that there was not enough input to strengthen capacity of the public health training universities regarding personnel and infrastructure. The institutions are often expected to seek and get by with short-term solutions.

The findings of this investigation complement those of earlier studies that attributed the lack of diversity in training programmes in LMICs to the multifaceted shortage of resources facing training institutions including faculty and support staff, funding, capacity to develop teaching material and curriculum, and teaching spaces [10, 11, 13, 47, 48]. This has been evident particularly in the context of University A, where the institution has fewer programmes than University B or University C due to lack of resources and support.

The successful introduction of the postgraduate programme on human resource management in University A suggests the important contribution that a south-south partnership can make. Literature on south-south cooperation reports on the fragile nature of such partnerships due to overreliance on funding from the North and capacity challenges to sustain partnership [34] despite their potential benefits in promoting Southern knowledge and experience, adaptability across partners, and non-hierarchical relationship [14, 49, 50] compared to north-south partnerships, whose success is undermined by “fundamentally unequal resource endowments and incentive structures” [51]. The literature on partnership further maintains that a host of factors pertaining to environment, membership, process and structure, communication, purpose, and resources determine success of partnerships [31, 32].

### Need for support and sustained engagement of programme champions

Literature emphasizes the value of backing programme champions if they are to make progress in fulfilling their roles, and that they need to have qualities like seniority, credibility, personality, and leadership [52–54]. Literature further highlights the significance of selecting the right personnel to execute the role of champion and support at their disposal [52, 55]. Our analysis also showed that programme champions’ success in enabling introduction of programme and building coherence across the different actors depends on seniority, sustained engagement, or availability of resource and institutional backing.

Programme champion’s role is as much political as technical. Their responsibility requires more than meeting curriculum approval standards, as they need to work towards securing endorsement from health ministries and other stakeholders to give the new programme legitimacy and required resources.

Taken together, the experiences of the three public health training universities resonate with other studies that recognize partnership and collaboration as key strategies for tackling complex challenges such as fostering innovation or improving performance [53, 54, 56]. Our analysis further shows that partnerships to introduce postgraduate programmes in public health training universities are fraught with contested interests and priorities around what is feasible or relevant; and potentiality of success in embedding and sustaining programme depends on alignment, coherence and harmonization of differences among the various players.

The findings from this study cannot be extrapolated to all public health training universities within the countries or the sub-Saharan region. The limited evidence suggests that universities within countries and in the region work with different set of actors, and have quite distinct experiences and relationships.

The study has two main limitations. First, determining impact and sustainability of new programmes, which are key but long-term aspects of capacity development, was not feasible within the limited period of this research. The focus of the research was thus limited to investigating processes, and short and medium-term outcomes of the intervention. Second, due to social desirability bias [57], and tensions between accountability and learning [58], participants may have been inhibited from fully disclosing failures that might reflect badly on themselves or their institution. We acknowledge the issue and strove to address it through long-term engagement, building trust and confidence with research participants to enable opportunities for open reflection and learning.

## Conclusions

Against the background of very limited human capacity and competition for this capacity, the process of introducing postgraduate programme in the three universities is a political as much as a technical undertaking influenced by multiple actors and agendas. The research shows that public health training universities are contested grounds among multiple actors (health ministry, education ministry, university, academics, and external development or training partners) vying for recognition or benefits, and influence over issues of programme feasibility, relevance or need. A successful introduction and further sustainability depend on alignment of interests and coherence in contribution of most of the actors, health ministries and universities in particular. Critical in the success of this south-south cooperation is support available to such initiatives; and how well programme champions are able to garner support for and ownership of programme locally. The paper argues that coherence and alignment are crucial to embed programmes, yet hard to achieve when capacity and resources are limited and contested.

## Data Availability

Not applicable.
